# Fasciite nécrosante monomicrobienne de la jambe due à l’*Acinetobacter baumannii* multirésistante chez un adulte sain: rapport d’un cas

**DOI:** 10.11604/pamj.2020.36.344.24614

**Published:** 2020-08-25

**Authors:** Jean Baptiste Ramampisendrahova, Rado Razafimahatratra, Gaëtan Duval Solofomalala

**Affiliations:** 1 Service de Chirurgie Orthopédique et Traumatologie, CHU Anosiala, Antananarivo, Madagascar

**Keywords:** Fasciite nécrosante, jambe, *Acinetobacter baumannii*, multirésistante, Necrotizing fasciitis, leg, Acinetobacter baumannii, multidrug-resistant

## Abstract

La fasciite nécrosante est une infection des tissus mous qui se propage rapidement et se caractérise par une nécrose étendue du fascia profond et superficiel. Il s’agit d’une infection polymicrobienne dans environ 70% des cas. L’infection monomicrobienne est généralement due aux streptocoques β-hémolytiques du groupe A. La fasciite nécrosante monomicrobienne due à l’Acinetobacter baumannii multirésistante est rare et survient généralement chez les patients immunodéprimés, ayant des antécédents médicaux, chez ces sujets l’infection est grave et mortelle à cause de la décompensation des tares sous-jacentes et le choc septique. La survenue de cette entité clinique chez le sujet sain est rare. Nous rapportons l’observation d’un homme âgé de 54 ans en bonne santé atteint d’une fasciite nécrosante monomicrobienne de la jambe gauche due à l’Acinetobacter baumannii multirésistante dont l’évolution était favorable après un débridement chirurgical étendu.

## Abstract (en)

Necrotizing fasciitis is a rapidly progressive soft tissue infection characterized by extensive necrosis in the deep and superficial fascia. This is a polymicrobial infection in about 70% of cases. Monomicrobian infection is usually caused by β-hemolytic group A streptococcus. Monomicrobian necrotizing fasciitis due to multidrug-resistant Acinetobacter baumannii is rare and usually occurs in immunocompromised patients with a medical history. In these subjects, the infection is severe and fatal due to the decompensation of the underlying defects and septic shock. The occurrence of this clinical disease in healthy subjects is rare. We report the case of a 54-year-old healthy man with monomicrobian necrotizing fasciitis of the left leg due to multidrug-resistant Acinetobacter baumannii; patient’s outcome was favorable after extensive surgical debridement.

## Introduction

La fasciite nécrosante est une infection des tissus mous qui se propage rapidement et se caractérise par une nécrose étendue du fascia profond et superficiel entraînant la dévascularisation et la nécrose des tissus associés [1]. Il s’agit d’une entité clinique rare avec environ 1000 cas par an aux États-Unis [2]. Même avec un traitement optimal, la fasciite nécrosante présage une morbidité importante et donne des taux de mortalité de 25% à 35% [2]. Elle constitue une véritable urgence médico-chirurgicale nécessitant un diagnostic précoce, un débridement chirurgical et une antibiothérapie à large spectre. Il s’agit d’une infection polymicrobienne dans environ 70% des cas avec diverses espèces de cocci à gram positif, de bactéries à gram négatif et des bactéries anaérobies; l’infection monomicrobienne est généralement causée par des streptocoques β-hémolytiques du groupe A [1]. La fasciite nécrosante monomicrobienne due à l’*Acinetobacter baumannii* est rare. Nous rapportons un cas d’une fasciite nécrosante monomicrobienne de la jambe due à l’*Acinetobacter baumannii* multirésistante chez un sujet sain.

## Patient et observation

**Figure 1:**
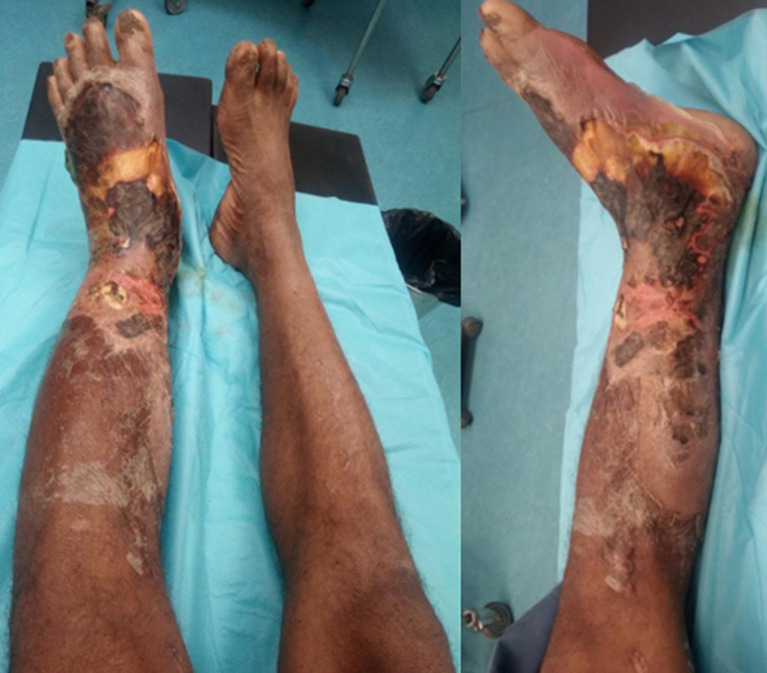
état clinique du patient à l’entrée aux urgences

Il s’agissait d’un homme de 54 ans, cultivateur, auparavant en bonne santé, sans antécédents médicaux ni chirurgicaux qui avait présenté une douleur et gonflement pied gauche, après une semaine d’une plaie traumatique négligée de la face dorsale de son pied, puis il avait fait une automédication par amoxicilline 500mg deux gélules matin-soir pendant cinq jours mais aucune amélioration. Au vingt-unième jour le patient s’est présenté dans un centre chirurgical privé car il avait constaté une aggravation de la lésion. Le chirurgien a indiqué une amputation d’emblée mais le patient a refusé cette amputation et il a rejoint le service des urgences du Centre Hospitalier Universitaire Anosiala Antananarivo. Le jour de la présentation aux urgences, le patient était conscient se plaignait d’une douleur intense de la jambe et du pied gauche, il était fébrile 38°C, ailleurs les signes vitaux étaient normaux. L’examen physique a révélé une odeur nauséabonde, induration et œdème du pied jusqu’à la moitié distale de la jambe, nécrose cutanée étendu de toute la partie dorsale du pied gauche, partie antérieure, médiale et latérale de la cheville gauche sans déficit sensitivo-moteur (Figure 1). L’examen biologique a montré une hyperleucocytose à 15,000/mm^3^ à prédominance neutrophile 82%, hémoglobine 13g/dl, une C-reactive protein (CRP) élevée à 160mg/l, créatinine 69μmol/l, ionogramme sanguin normale avec sodium 135mmol/l, glycémie 5,3g/l. Le score LRINEC (Laboratory Risk Indicator for Necrotizing Fasciitis) était à 6 (Tableau 1).

**Tableau 1:** score biologique de

Paramètres	Valeurs	Score	Score de notre patient
CRP (mg/l)	<150	0	
	>150	4	4
Globule blanc (cellules/mm^3^)	<15 000	0	
	15-25 000	1	1
	>25 000	2	
Hémoglobine (g/dl)	>13,5	0	
	11-13,5	1	1
	<11	2	
Sodium (mmol/l)	>135	0	0
	<135	2	
Créatinine (mg/dl)	<1,6	0	0
	>1,6	2	
Glycémie (mg/dl)	<180	0	0
	>180	1	

Risque faible: score ≤5, probabilité <50%; Risque intermédiaire: score 6-7, Probabilité 50-75% Risque élevé: ≥8, probabilité >75%

**Figure 2:**
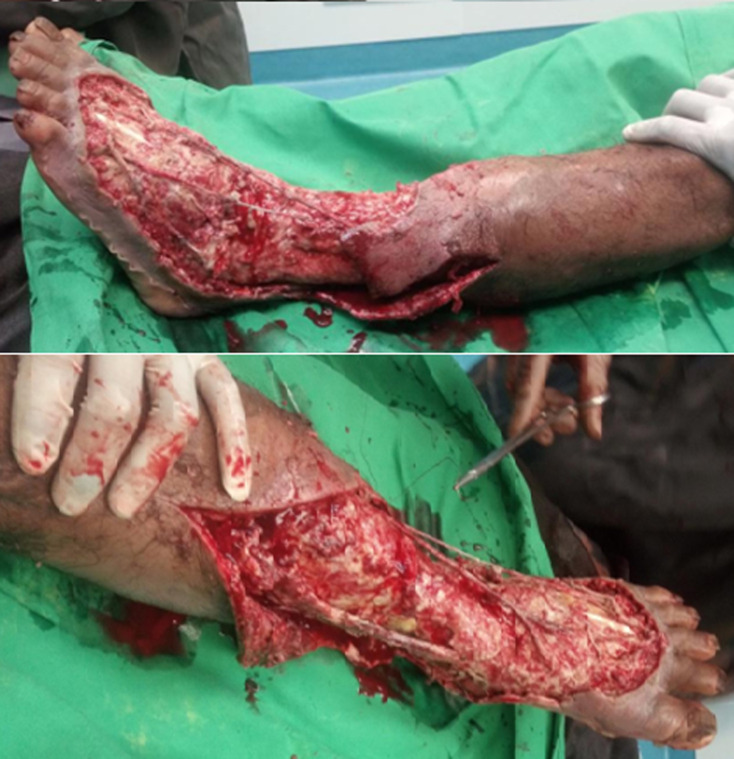
état après débridement chirurgical

**Figure 3:**
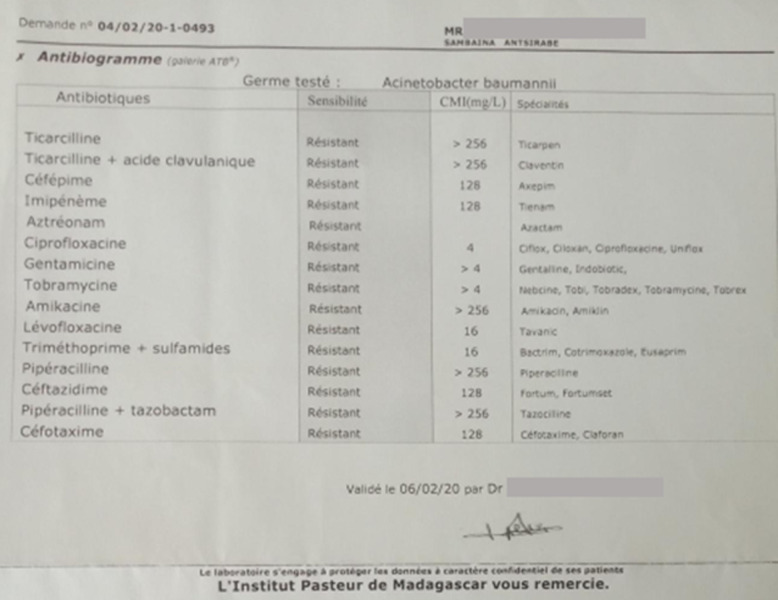
résultat bactériologique et antibiogramme

**Figure 4:**
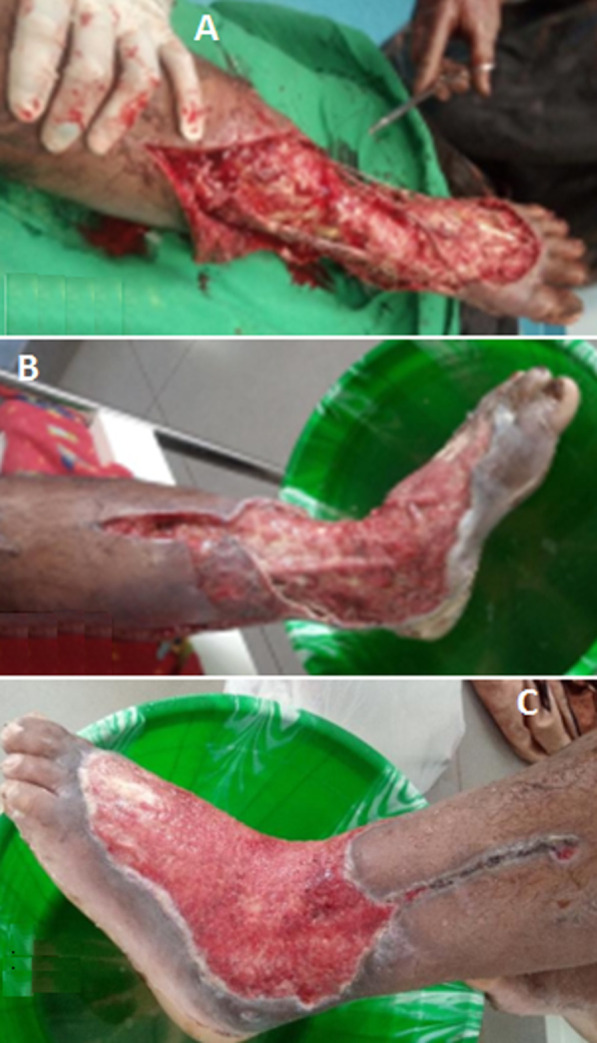
évolution de la plaie

L’échodoppler des vaisseaux du membre inférieur était normal, la radiographie n’a pas trouvé des signes d’atteinte osseuse. Le patient a bénéficié une intervention chirurgicale le même jour de son admission. Au cours du débridement, une nécrose sévère s’est étendue du pied gauche à la jambe gauche; par conséquent, un vaste débridement était nécessaire, tous les tissus dévitalisés étaient retirés du dos du pied de la cheville et de la moitié inférieure de la jambe (Figure 2). Le fascia a été échantillonné pendant la procédure et envoyé à l’Institut Pasteur de Madagascar pour examen bactériologique et antibiogramme. Une antibiothérapie parentérale probabiliste, association céftriaxone 2g par jour, ciprofloxacine 400mg par jour et gentamicine 240mg par jour était effectuée en attendant le résultat bactériologique. Le pansement était réalisé tous les jours dès le lendemain de l’intervention avec un bain au Dakin pendant 15min avant tous les pansements. Le germe isolé était l’*Acinetobacter baumannii* multirésistante (Figure 3). Comme aucun antibiotique n’était pas sensible et on a décidé d’arrêter tous les antibiotiques et de continuer le pansement journalier et le bain au Dakin. L’évolution était favorable, la plaie était propre avec les tissus mous bien bourgeonnés (Figure 4). Après un mois d’hospitalisation, une greffe cutanée a été organisée, le bilan bactériologique préopératoire avait montré le même germe. La décision d’une cicatrisation dirigée a été prise en continuant le même principe de pansement avec une fréquence tous les deux jours. Le patient est sorti de l’hôpital et continuait en externe son pansement avec un contrôle tous les 15 jours dans le service.

## Discussion

L’infection nécrosante du tissu mou a été décrite pour la première fois par Hippocrate au quinzième siècle avant Jésus Christ, il l’a décrit comme une complication d’une infection streptococcique aiguë [1,2]. La fasciite nécrosante est une entité clinique extrêmement rare, avec environ 1000 cas par an aux États-Unis; il semble que cette incidence ait augmenté à cause peut être du résultat d’une plus grande prise de conscience du problème conduisant à des taux plus élevés de déclaration, à une virulence bactérienne accrue, à une résistance accrue aux antimicrobiens [2]. Il n’y a pas de prédilection pour l’âge ou le sexe, mais des taux plus élevés de la fasciite nécrosante étaient observés chez les patients obèses, diabétiques et immunodéprimés, ainsi que chez les alcooliques et les patients atteints d’une maladie vasculaire périphérique, elle relativement rare chez l’enfant. Cependant, la fasciite nécrosante peut survenir chez les sujets jeunes en bonne santé, sans aucun de ces facteurs prédisposants [3], le cas de notre patient qui était un homme adulte jeune sain qui avait un traumatisme du pied gauche négligé et maltraité. L’induration et œdème du pied jusqu’à la moitié distale de la jambe, nécrose cutanée étendu de toute la partie dorsale du pied gauche, partie antérieure, médiale et latérale de la cheville gauche sont les signes cliniques observés dans notre cas. Les premiers signes et symptômes de la fasciite nécrosante sont identiques à ceux observés avec la cellulite ou les abcès, ce qui rend potentiellement le diagnostic correct difficile en général, l’érythème, la douleur au-delà des marges d’infection évidente, l’enflure et la fièvre sont les résultats les plus courants de l’examen physique [2].

La présentation clinique variera en fonction du germe pathogène responsable, ainsi que de la région anatomique et de la profondeur de l’infection. Livingstone *et al*. [4] ont rapporté un cas d’un homme de 56 ans s’est manifesté cliniquement par un érythème diffus et une enflure au membre inférieur droit s’étendant jusqu’à la face médiale de la cuisse droite. Xu LQ *et al*. [1] ont trouvé dans la fasciite due au *Staphylococcus aureus* une décoloration localisée rouge-violacé sur les deux membres inférieurs. Tsai YH *et al*. [5] dans leur cas sur la fasciite nécrosante monomicrobienne causée par *Aeromonas hydrophila* et *Klebsiella pneumoniae* ont rapporté que l’enflure du membre impliqué avec des lésions cutanées bulleuses œdémateuses, inégales, érythémateuses et hémorragiques étaient les signes cliniques observés moment de l’admission aux urgences. Le score LRINEC (Laboratory Risk Indicator for Necrotizing Fasciitis), un outil pour distinguer la fasciite nécrosante des autres infections des tissus mous. Les scores ≥6 se sont révélés avoir une valeur prédictive positive de 92% et une valeur prédictive négative de 96%. Notre patient avait un score de six [2]. Bien que ce score soit largement utilisé, son utilisation est limitée lorsque des états inflammatoires concurrents sont présents. Ainsi, Hakkarainen *et al*. [2] ont pensé qu’il ne doit pas être utilisé que dans le contexte d’une présentation clinique plus large et doit être interprété avec prudence; les décisions de traitement ne doivent pas être basées uniquement sur le score LRINEC.

Selon le microorganisme impliqué, les fasciites nécrosantes sont classées en quatre types: le type I est classiquement polymicrobienne et diverses espèces de cocci à gram positif, des bactéries à gram négatif et des bactéries anaérobies sont généralement isolées; les infections type II est généralement monomicrobienne, généralement causée par des streptocoques β-hémolytiques du groupe A seuls ou en combinaison avec des espèces de staphylocoques; le type III décrit une infection spécifique causée par *Vibrio vulnificus* marin et le type IV est causé par une infection fongique dont les espèces les plus courantes étant *Candida* spp. [1,2]. La fasciite nécrosante monomicrobienne due à l’*Acinetobacter baumannii* multirésistante chez un sujet sain comme le cas de notre patient est rare. Tous les cas publiés étaient survenus chez le sujet immunodéprimé, ayant des maladies sous-jacentes. Ali A *et al*. [6] ont rapporté un cas d’un homme de 41 ans, obèse ayant des antécédents médicaux de cirrhose du foie chez un ancien alcoolique, d’hépatite C.

Matthews *et al*. [7] ont rapporté un cas d’une femme immunodéprimée de 37 ans qui avait des antécédents médicaux complexes plus connus pour le lupus érythémateux systémique. Le traitement consiste à un débridement total, tous les tissus nécrotiques doivent être excisés et les échantillons sont envoyés pour des études de culture immédiatement après la procédure. La perte cutanée étendue nécessite des greffes de peau [3]. Nous avons décidé de faire une cicatrisation dirigée, car le bilan bactériologique préopératoire pour la greffe cutanée de la plaie a révélé positif avec le même germe qui faisait la contre-indication de la greffe cutanée. Livingstone *et al*. [4] a utilisé la thérapie à pression négative en poste opératoire immédiate puis greffe cutanée après quatre semaines. L’évolution de notre patient était favorable, par contre, chez le sujet immunodéprimé ayant des antécédents médicaux, la fasciite nécrosante due à *l’Acinetobacter baumannii* est grave. Matthews *et al*. [7] ont rapporté un cas décédé. Nehme A *et al*. [8] ont rapporté aussi un cas décédé à cause d’un choc septique après une deuxième intervention pour une amputation secondaire du fait de l’extension de la nécrose après quatre jours du premier débridement.

## Conclusion

La fasciite nécrosante monomicrobienne due à *l’Acinetobacter baumannii* multirésistante est une entité clinique rare, mais il semble actuellement que son incidence ait augmenté. Elle survient généralement chez les patients immunodéprimés ayant des antécédents médicaux, chez ces sujets l’infection est grave et mortelle à cause décompensation des tares sous-jacentes et le choc septique. La survenue de cette entité clinique chez le sujet sain comme notre cas est rare mais d’évolution favorable après un débridement chirurgical étendu.

## Remerciements

Je suis extrêmement reconnaissant à toute l’équipe de Chirurgie, des Urgences et du Bloc du CHU Anosiala, sans leur soutien, il n’aurait été pas possible de mener cette étude.
